# Clinical Reflections

**Published:** 1883-06

**Authors:** Ad. Lippe

**Affiliations:** Philadelphia


### CLINICAL REFLECTIONS.

Ad. Lippe, M. D., Philadelphia.

Mr. W., æt. 45, was taken sick on the 11th of April, 1883. For the better understanding of the case, a very short report of his former sickness will be given. Mr. W. was a robust, healthy child. When about twenty years old he took a great deal of Mercury, which apparently cured the disorder for which it was administered. He suffered ever since from fissures and frequent inflammations of the throat. The fissures were operated on several times—of course, unsuccessfully. In 1876 he was taken down with typhus fever, and was treated in the ordinary manner. A few years previous to this attack he had abandoned the evil habit of using wines and all other liquors to excess, but continued to use tobacco to excess. Since the fall of 1876 he has been under homœopathic treatment; his health had much improved; his attacks of tonsilitis and occasional ulcerations of the throat had almost entirely subsided. Apparently, he was in robust health, but at times he would suffer from a violent stomach cough, and at such times he always coughed most severely after breakfast and until he vomited up all he had eaten.

For a week he had felt badly; had taken a few doses of Nux vom., when he had a severe fall, slipping head-foremost on a flight of stairs. Complaining of feeling as if he had strained himself severely from an effort to grasp the banister of the stairs to regain his position, he had taken that evening Arnica and received the next morning, April 10th, Rhus tox. In the night of the 11th of April he had a chill, but arose in the morning of the 12th, staying in the house; had no desire to eat; that night he had three distinct severe chills, followed by a high fever; found him in the morning of the 13th very weak; had a sleepless, very restless night; intense thirst, tongue dry and black, lips black, great pain in the small of the back, and headache. He now received Arsenicum—one dose in the morning. The restlessness and the pain in the back ceased gradually, and on the 14th I found he had spent a sleepless night, but was now lying perfectly quiet, more so because every movement caused him great pain, especially in the now swollen joints; the tongue was no longer black but dry; intense thirst and fever continued; occasionally he suffered from cough, which caused him to retch violently, when he would vomit up some very tough, stringy white mucus; skin dry; urine scanty and dark. Gave him in the morning one dose of Bryonia, and finding him no better in the evening, the retching attacks having become more frequent, I gave him Bryonia in water every two hours. On the 15th I found in the morning that he had had another sleepless night, the thirst, fever, retching continued; the muscular pains were worse; his taste was sour; the skin was moist and the urinary secretions abundant; urine natural; no discharge from the bowels since the 11th; no food had been taken since the 11th; throat sore, highly inflamed; his headache was very severe and his longing for sleep very great; gave a dose of Belladonna.

On the 16th I found he had another sleepless night; the muscular pains were much worse on motion; his throat more sore; the retching much more frequent and the quantity of the stringy, white mucus much increased; skin moist; thirst continued great for cold water; the tongue more furred but less dry. Gave Kali bichr., which had on former occasions with similar sore throat always relieved him, but on the 17th I found him no better; a sleepless night; the retching much more frequent, causing much soreness in the stomach; more abundant secretions of normal urine and some perspiration; thirst continued; fever less. No medicine. On the 18th he was no better, but the perspiration and urinary secretions more profuse; the fever much less; the rheumatic pains very severe; feels sore all over; no sleep; less thirst. No medicine, but was sent for at 11 P. M. The retching spells with cough and raising of viscid mucus had become much more frequent in the evening, and during one of these attacks he was seized with an excruciating pain under the right lower ribs, which was aggravated on breathing and by the least motion, which made him cry out with pain. As I entered the sick-room, I found him bending over a basin at the side of his bed coughing and retching, bringing up a large quantity of mucus with very small streaks of blood in it and violently crying out with the pain in his side. One dose of Arnica$^{cm}$ (Fincke) was given, a few pellets on the tongue. A few minutes later he had another attack of coughing and retching, but less severe than the former one, and still ten minutes later he had a very short attack and now laid quiet—the pain in his side became endurable. I left him at 1 A. M., confident that Arnica was doing its work and predicted that he would go to sleep at 2 A. M., which he did. On the 19th I found him much better in every respect—no cough or retching since I left him and none since this 8th day of May. He now slept well; his appetite gradually returned and he was kept on farinaceous food till the 24th, when he relished animal food. All he complained of on the 24th was great itching all over the body, for which he received one dose of Sulphur$^{cm}$ (Fincke). On the 28th he rode out; on the 29th he walked out, and left for the seashore on the 30th, where he rapidly gained his accustomed strength. He has "*forever*" given up the use of tobacco.

*Comments:* The first question *in our days* is, What sort of a disease was this—what was "The Diagnosis"? As everything, from a child down to the products of disease, must have a name, we should suggest to call this case a gastric-rheumatic-nervous-fever. Others may see in it a malarial out-cropping, and, if so, insist upon it that Pyrogen, if given in a very, very high potency just at first, would have cured the patient on sight. Or another celebrity, who has transferred his skill as a diagnostician, which he acquired among his former allopathic teachers and colleagues, to our school, and who really advanced without reflection and fell into a trap set for him, which trap was set in a bottle-washing establishment, the inscription on whose door reads: "*Morbific products if highly potentized* will cure the disease which produces them." A reflecting mind would see through such a thin disguise and at once conclude that said bottle-washer had set up the untenable doctrines that a morbific-product becomes a curative agent *when highly potentized*, while Homœopathicians hold on to first principles, viz.: That medicinal substances must first be proved on the healthy organism; that thereby their sick-making properties may be discovered and so ascertained, may enable the true healer to apply them for the cure of the sick under the eternal law of the Similars. Furthermore, an experiment tried by the unlucky man who progresses first and reflects afterward will prove to be a disgraceful failure. As a close observer and having a great propensity to investigate all new discoveries, we once stumbled across one of these great diagnosticians, who promptly diagnosticated "syphilis," and Syphilinum was just as promptly administered, and the patient, believing herself under homœopathic treatment, grew worse; the tumor on the left labium major grew larger and more painful, was finally operated upon, and was found to be "a cancer." Another one of these bottle-washers picked up a homœopathic patient at a watering-place and without fully examining the case, and of course ignorant of the steadily progressing improvement of the young lady's health, promptly diagnosticated tuberculosis, and as promptly administered Tuberculinum. The patient grew steadily worse and died of tuberculosis. Now we did suggest a diagnosis in the case but did not, could not, utilize it or any other diagnosis, in the treatment of the case; that is the prerogative of all other schools, allopathic, eclectic, and isopathic. The pathology of diseases will be written by a future generation, and these men will be progressive homœopathicians, they will see in this case merely a salutary revival of previously palliated diseases.

The night of the seventh day was the critical time in the case above related, and the situation of the patient was both painful and critical. At just such times do we find the desired aid in strictly following "the master;" this is the *only* hope we have. Thoughtless and spineless pretenders demand that on just such occasions humanitarianism demands the application of boasted, but often failing, palliatives, such as are hypodermic injections of Morphia, etc.

The true healer's true humanitarianism demands the strictest possible adherence to principles. The homœopathician will find that in this case Arnica covered all the symptoms of the patient. Almost all of them are to be found in the pathogenesis of that remedy, and a few could easily be found to be similar by analogy. The disease, which had fully developed itself for seven days, now showed its true inwardness through discernible symptoms. The similar remedy was applied in a single dose, and the recovery was observed as is usual in similar forms of diseases.

The lesson attached to this case and to similarly treated cases, is that we must, as consistent homœopathicians, as humanitarians, and as true healers, remain faithful to our professed principles, and reject all possible "substitutes." When we find upon calm investigation that such substitutes are not in harmony with the well-proven and correct tenets of our school, it is our duty to reject them, and, advancing in the right direction, fully expose the fallacy of all such substitutes and innovations.

